# Patient selection for milk and egg ladders using a food ladder safety checklist

**DOI:** 10.1186/s13223-022-00696-w

**Published:** 2022-06-12

**Authors:** Gilbert T. Chua, Edmond S. Chan, Joanne Yeung, Scott B. Cameron, Lianne Soller, Brock A. Williams, Alanna Chomyn, Timothy K. Vander Leek, Elissa M. Abrams, Raymond Mak, Tiffany Wong

**Affiliations:** 1 Department of Paediatrics and Adolescent Medicine, Li Ka Shing Faculty of Medicine, The University of Hong Kong, 1/F, New Clinical Building, Queen Mary Hospital, 102 Pokfulam Road, Hong Kong,, SAR China; 2 Department of Paediatrics and Adolescent Medicine, The Hong Kong Children’s Hospital, Hong Kong, SAR China; 3 Department of Paediatrics, The University of Hong Kong-Shenzhen Hospital, Shenzhen, China; 4 Division of Allergy and Immunology, Department of Pediatrics, University of British Columbia, Vancouver, BC Canada; 5 British Columbia Children’s Hospital Research Institute, Vancouver, BC Canada; 6 Community Allergy Clinic, Victoria, BC Canada; 7 Food, Nutrition, and Health, Faculty of Land and Food Systems, University of British Columbia, Vancouver, BC Canada; 8 Department of Pediatrics, Pediatric Allergy & Asthma, University of Alberta, Edmonton, AB Canada; 9 Department of Pediatrics, Section of Allergy and Clinical Immunology, University of Manitoba, Winnipeg, Canada

**Keywords:** Food ladders, Milk allergy, Egg allergy, Safety

## Abstract

A food ladder is a form of home-based dietary advancement therapy that gradually increases exposure to an allergenic food through the gradual introduction of egg or milk containing food with increasing quantity and allergenicity from extensively heated forms, such as baked goods, to less processed products. While widely considered safe, the food ladder is not risk-free and most of the egg and milk ladder studies only included preschoolers with mild egg and milk allergies, and with no or well-controlled asthma. We propose a Food Ladder Safety Checklist to assist with patient selection using “4 A's” based on available evidence for food ladders, including Age, active or poorly controlled Asthma, history of Anaphylaxis, and Adherence.

**Supplementary Information:**

The online version contains supplementary material available at 10.1186/s13223-022-00696-w.

## To the Editor,

In May 2021, a young girl in Ontario, Canada, with a history of milk allergy and long-standing asthma, passed away while undergoing a therapy that some have described as a milk ladder, although media reports suggest she did not increase beyond tiny amounts of milk-containing muffin [1, 2]. This tragic incident deeply saddens the allergy community, and underscores the need for careful patient selection and close monitoring of patients undergoing all forms of dietary advancement therapy. This article discusses the benefits, risks, and precautions of food ladders as a form of dietary advancement therapy, as well as how to help our patients and families decide whether ladders are an appropriate treatment option through a shared decision-making process. We propose a Food Ladder Safety Checklist to assist with patient selection.

## What is a food ladder?

A food ladder is a form of home-based dietary advancement therapy that gradually increases exposure to an allergenic food. Egg and milk ladders are the two typical forms of food ladders used clinically. The goal of the food ladder is to facilitate the development of natural tolerance through the gradual introduction of egg or milk containing food with increasing quantity and allergenicity through different cooking processes, typically with gradual progression from baked products (e.g., biscuits, muffin), to well-cooked forms (e.g., pancakes, waffles, hard-boiled eggs) and finally to less processed products (e.g., fresh mousse, fresh ice cream) [3]. It has been widely used in Europe and was initially designed to manage non-IgE-mediated food allergies [4, 5]. Subsequently, application of the food ladder has been extrapolated to management of IgE-mediated milk and egg allergy, which has been generally safe and effective [6–8].

## What are the benefits and risks of the food ladder?

The primary benefits of home-based treatments such as milk and egg ladders include the demedicalization of food (by providing a structured approach which still allows flexibility in options and pace at which individuals proceed), and reduction in health care utilization. For example, they allow practitioners the ability to allocate limited in-person appointments for oral food challenges and oral immunotherapy to other patients who are too high-risk for home-based treatments. While some practitioners offer a starting dose in the office, home-based therapies typically involve fewer in-office visits. Studies have shown that even home-based oral immunotherapy for IgE-mediated food allergy can be feasible and safe with very carefully selected patients [9, 10], which offers hope for facilitating early commencement of dietary advancement therapy where resources are limited with long waiting times, especially during the COVID-19 pandemic when there were limited non-emergency elective services and lack of regular in-office visits [11, 12].

Ball et al. retrospectively studied 86 children with mild milk allergies who started home-based milk introduction between 8 to 33 months of age; 68 of 86 subjects (79.1%) reached the top of the milk ladder at the two-year mark and two additional subjects tolerated all dairy products at the fourth review. None developed anaphylaxis or required epinephrine autoinjector [6]. Gotesdyner et al. studied 39 children under two years old with mild egg allergy and treated them using a structured graduated exposure protocol, and compared with a matched group of 80 children who were advised to strictly avoid egg at least until two years old or earlier natural resolution and followed to a median age of 69 months. The age of egg allergy resolution in the treatment group was significantly younger than the control group (median age 24 months vs. 78 months, p < 0.001), and 82% of children in the treatment group were able to tolerate lightly cooked eggs, versus 54% in the control group (p = 0.001) [8]. Thomas et al. retrospectively reviewed 98 children with a median age of 40 months with mild egg allergy and were managed with egg ladder. 43% were managed with an egg ladder over an average of 15.5 months. Only two children had severe reactions, and one required adrenaline; those with severe reactions resumed the ladder and progressed to the last two steps (lightly cooked whole egg or raw egg) successfully. A high proportion (78.7%) of the parents felt satisfied or very satisfied with the egg ladder [7] (Table 1).

**Table 1:** Summary of studies using egg and milk ladders to treat IgE-mediated egg and milk allergy

Author/Year/Study Type/Ladder	Inclusion criteria	Exclusion criteria	No of subjects in active treatment group	Age	Main outcomes	% of any adverse reactions	Anaphylaxis	Risk factors for anaphylaxis
Ball et al./2019 [6] Retrospective chart review/Milk Ladder	IgE-mediated cow's milk allergy based on clinical history and positive SPT	– Cow's milk allergic reactions occurring with trace baked milk ingestion – Allergic reactions involving the respiratory or cardiovascular systems – History of recurrent wheeze – SPT > 8 mm	86	Median 13 months (range 8—33 months)	– 68 subjects (79.1%) reached the top of the milk ladder by the two-year mark – 2 subjects tolerated all dairy products at the fourth review	80%	None	N/A
Gotesdyner et al./2019 [8] Case Control Study/Egg Ladder	-Children < 2 years old -IgE‐mediated egg allergy diagnosed by OFC or by positive SPT and/or positive IgE along with a clinical history of an immediate allergic reaction after exposure to cooked or fried eggs in the past year	Children with a history of allergic reaction to baked egg were excluded from the control group	39	Median 16 months (IQR: 14—19 months)	– Significantly younger age of allergen resolution in the treatment group than the control group (median age 24 months vs. 78 months, p < 0.001) – 82% of children in the treatment group were able to tolerate lightly cooked eggs, versus 54% in the control group (p = 0.001)	23%	One patient developed anaphylaxis (rash and vomiting) and EpiPen was given. The protocol was stopped and the child continued with egg avoidance	Not mentioned
Thomas et al./2021 [7] Retrospective study/Egg Ladder	– Single food allergy – History convincing of IgE-mediated egg allergy – Mild or no eczema – No or well-controlled asthma Written action plan for food allergy management and education provided	History of anaphylaxis to any food containing egg or a non-IgE-mediated egg allergy	47	Mean age 40 months (IQR: 12–60 months)	43% were able to complete the egg ladder over an average of 15.5 months	59.60%	Two patients had severe reaction (by parent report). One was treated with adrenaline	Not mentioned

*IgE* Immunoglobulin E, *IQR* interquartile range, *OFC* oral food challenge, *SPT* skin prick testing

## Patient selection for milk and egg-allergic patients

Like any other dietary advancement therapy, the food ladder should be considered a form of oral immunotherapy and therefore not risk-free. Most of the egg and milk ladder studies only included preschoolers with mild egg and milk allergies without a history of anaphylaxis (who have a high likelihood of outgrowing their food allergy), together with well-controlled or no asthma, and families who could follow food allergy management and anaphylaxis action plans [6–8] (Table 1). Other studies limited the inclusion of egg and milk allergic subjects who had passed a baseline oral food challenge to baked egg or milk, and used the food ladder as a tool to facilitate the desensitization process [5].

Emerging real-world evidence has demonstrated that performing oral immunotherapy for food allergy early, especially during infancy and preschool age (< 6 years old), is significantly safer, more effective, and more likely to result in sustained unresponsiveness, compared to starting later in older children [13–19]. It is also known that infants and toddlers have fewer allergic reactions involving the respiratory, cardiovascular, and neurological systems compared to older children [20–22]. Evidence on the safety of using food ladders to desensitize older children and adolescents with persistent allergy to baked egg and milk is also lacking. Therefore, patients with persistent higher-risk phenotypes such as those with older age (> 6 years old), previous anaphylactic reactions to extensively baked forms of food (especially involving the respiratory and cardiovascular systems), a prior history of allergic reaction at a very low trigger threshold, poor asthma control, or psychosocial factors (e.g. families unable to adhere to instructions or follow-up) are not suitable candidates for food ladders [6, 7].

## Pre-requisites for administering food ladders safely

The food ladder should be administered by well-trained and experienced healthcare professionals with the necessary expertise and experience in food allergy and anaphylaxis management, performance of oral food challenges, and careful selection of patients for food immunotherapy [2]. Patient informed consent should be obtained, and patients should be aware of cofactors that could lower reaction threshold while on any dietary advancement therapy, including febrile illnesses, exercise, hot baths, dosing on an empty stomach, and an increase in total allergen exposure such as dust mite and pollen [23, 24]. Allergists should be readily available to address patient concerns and reactions,and to ensure families are confident and competent at treating anaphylaxis before offering ladders. Allergists should also be aware of conditions where using food ladders as a dietary advancement therapy could be risky and less effective. On-going close follow-up with the allergist administering the food ladders is essential to ensure the safety, ongoing understanding of the procedures and to ensure new unexpected risk factors (e.g. loss of asthma control) have arisen. We propose a Food Ladder Safety Checklist to assist with patient selection using “4 A’s” based on available evidence for food ladders, including **A**ge, active or poorly controlled **A**sthma, history of **A**naphylaxis, and **A**dherence (Additional file 1). Allergists may decline or delay offering food ladders while optimizing any modifiable factors, such as asthma, or opt for an alternative dietary advancement therapy such as oral immunotherapy (OIT).

## A shared decision-making process to making the most suitable choice

Shared decision-making (SDM) refers to the process by which patients play an active role in managing their health [25] It is also a bidirectional conversation that incorporates pros and cons of approaches and integrates patient preferences into decision making. This is different from informed consent, in which patients only agree or disagree with a treatment option. SDM involves three steps: (i) creating choice awareness but providing an unbiased list of options, (ii) discussing options based on clinical relevance and current medical evidence, and (iii) discussing patient preferences, i.e., “what matters most” to the patient. It is essential to clarify goals and expectations of treatment, experience with previous management strategies, and possible fears. In the context of food allergy, it is important that the allergist provides different options to patients and families [25]. For example, if a patient has multiple food allergies, an option of milk or egg OIT instead of ladders could be incorporated as part of a multiple food OIT protocol, which has also been shown to be safe and effective [26] Ultimately, for patients with identified contraindications such as uncontrolled asthma, or where dose adherence would be unlikely, strict avoidance while carrying an epinephrine autoinjector and future reassessment for spontaneous resolution might be a better option [27].

## Conclusion

Milk and egg ladders are safe and effective dietary advancement therapies, in patients who have a high likelihood of outgrowing their milk and egg allergies. Nevertheless, any form of dietary advancement therapy carries a risk of allergic reaction, including anaphylaxis, as these patients are still allergic to milk and egg at baseline. Careful attention needs to be paid to proper patient selection and managing allergic comorbidities such as asthma, prior to initiating a milk or egg ladder, and a food ladder safety checklist can assist with patient selection. Despite recent media reports, the risk of death with milk/egg ladders or food immunotherapy in carefully selected patients is extremely remote, and does not exceed the risk of death from avoidance or other forms of allergen immunotherapy such as subcutaneous immunotherapy with aeroallergens [2].

## Supplementary Information

**Additional file 1. **The food ladder safety checklist.

## Data Availability

Not applicable.

## Abbreviations

IgE: Immunoglobulin E
IQR: Interquartile range
OFC: Oral food challenge
OIT: Oral immunotherapy
SDM: Shared decision-making
SPT: Skin prick testing

## References

[1] Mondello W. Girl with milk allergy dies of severe reaction related to desensitization 2021. https://www.allergicliving.com/2021/12/20/girl-with-milk-allergy-dies-of-severe-reaction-related-to-desensitization/.

[2] CSACI/BSACI Statement on OIT 2022. https://www.csaci.ca/wp-content/uploads/2022/01/CSACI-_-BSACI-Statement-on-OIT.pdf.

[3] Leech SC, Ewan PW, Skypala IJ, Brathwaite N, Erlewyn-Lajeunesse M, Heath S (2021). BSACI 2021 guideline for the management of egg allergy. Clin Exp Allergy.

[4] Venter C, Brown T, Shah N, Walsh J, Fox AT (2013). Diagnosis and management of non-IgE-mediated cow's milk allergy in infancy—a UK primary care practical guide. Clin Transl Allergy.

[5] Lambert R, Grimshaw KEC, Ellis B, Jaitly J, Roberts G (2017). Evidence that eating baked egg or milk influences egg or milk allergy resolution: a systematic review. Clin Exp Allergy.

[6] Ball HB, Luyt D (2019). Home-based cow's milk reintroduction using a milk ladder in children less than 3 years old with IgE-mediated cow's milk allergy. Clin Exp Allergy.

[7] Thomas L, Belcher J, Phillips R, Preece K, Bhatia R (2021). Use of an egg ladder for home egg introduction in children with IgE-mediated egg allergy. Pediatr Allergy Immunol.

[8] Gotesdyner L, Zeldin Y, Machnes Maayan D, Efron A, Stauber T, Maoz Segal R (2019). A structured graduated protocol with heat denatured eggs in the treatment of egg allergy. Pediatr Allergy Immunol.

[9] Chua GT, Chan ES, Soller L, Cook VE, Vander Leek TK, Mak R (2021). Home-based peanut oral immunotherapy for low-risk peanut-allergic preschoolers during the COVID-19 pandemic and beyond. Front Allergy..

[10] Garvey AA, O'Sullivan D, Hourihane JO (2017). Home-based induction of sustained unresponsiveness in children with mild reactions to high doses of peanut. J Allergy Clin Immunol Pract.

[11] Mack DP, Chan ES, Shaker M, Abrams EM, Wang J, Fleischer DM (2020). Novel approaches to food allergy management during COVID-19 inspire long-term change. J Allergy Clin Immunol Pract.

[12] Chan YT, Ho HK, Lai CK, Lau CS, Lau YL, Lee TH (2015). Allergy in Hong Kong: an unmet need in service provision and training. Hong Kong Med J.

[13] Soller L, Abrams EM, Carr S, Kapur S, Rex GA, Leo S (2019). First real-world safety analysis of preschool peanut oral immunotherapy. J Allergy Clin Immunol Pract.

[14] Soller L, Abrams EM, Carr S, Kapur S, Rex GA, Leo S (2021). First real-world effectiveness analysis of preschool peanut oral immunotherapy. J Allergy Clin Immunol Pract.

[15] Soller L, Carr S, Kapur S, Rex GA, McHenry M, Cook VE (2021). Real-world peanut OIT in infants may be safer than non-infant preschool OIT and equally effective. J Allergy Clin Immunol Pract.

[16] Jones SM, Kim EH, Nadeau KC, Nowak-Wegrzyn A, Wood RA, Sampson HA (2022). Efficacy and safety of oral immunotherapy in children aged 1–3 years with peanut allergy (the Immune Tolerance Network IMPACT trial): a randomised placebo-controlled study. The Lancet.

[17] Vickery BP, Vereda A, Casale TB, Beyer K, du Toit G, Hourihane JO (2018). AR101 oral immunotherapy for peanut allergy. N Engl J Med.

[18] Chu DK, Wood RA, French S, Fiocchi A, Jordana M, Waserman S (2019). Oral immunotherapy for peanut allergy (PACE): a systematic review and meta-analysis of efficacy and safety. Lancet.

[19] Vickery BP, Berglund JP, Burk CM, Fine JP, Kim EH, Kim JI (2017). Early oral immunotherapy in peanut-allergic preschool children is safe and highly effective. J Allergy Clin Immunol.

[20] Ko J, Zhu S, Alabaster A, Wang J, Sax DR (2020). Prehospital treatment and emergency department outcomes in young children with food allergy. J Allergy Clin Immunol Pract.

[21] Samady W, Trainor J, Smith B, Gupta R (2018). Food-induced anaphylaxis in infants and children. Ann Allergy Asthma Immunol.

[22] Kennedy K, Alfaro MKC, Spergel ZC, Dorris SL, Spergel JM, Capucilli P (2021). Differences in oral food challenge reaction severity based on increasing age in a pediatric population. Ann Allergy Asthma Immunol.

[23] Wasserman RL, Factor J, Windom HH, Abrams EM, Begin P, Chan ES (2021). An approach to the office-based practice of food oral immunotherapy. J Allergy Clin Immunol Pract.

[24] Chua GT, Chan ES, Soller L, Cameron SB (2021). Grass pollen allergy as an anaphylaxis cofactor during peanut oral immunotherapy. Ann Allergy Asthma Immunol.

[25] Graham F, Mack DP, Bégin P (2021). Practical challenges in oral immunotherapy resolved through patient-centered care. Allergy Asthma Clin Immunol.

[26] Eapen AA, Lavery WJ, Siddiqui JS, Lierl MB (2019). Oral immunotherapy for multiple foods in a pediatric allergy clinic setting. Ann Allergy Asthma Immunol.

[27] Foong R-X, Santos AF (2021). Biomarkers of diagnosis and resolution of food allergy. Pediatr Allergy Immunol.

