# Supplementary material for: Patient selection for milk and egg ladders using a food ladder safety checklist
Source: Allergy Asthma Clin Immunol. 2022 Jun 12;18:51. doi: 10.1186/s13223-022-00696-w (PMC9188637; doi:10.1186/s13223-022-00696-w)
Supplement: Supplementary file 1 — Additional file 1. The food ladder safety checklist. [file 13223_2022_696_MOESM1_ESM.docx]

**The Food Ladder Safety Checklist**

**Which children might not be a good fit for a milk or egg ladder? The 4 A’s**

- **Age:** Children 6 years or older
- **Asthma: Severe or not well-controlled (1 or more of the following):**
  - **Visit to the emergency room or a hospital stay within the last 6 months**
  - **Need for oral corticosteroids within the last 6 months**
  - **Nighttime symptoms, especially if needing rescue inhaler**
  - **Asthma symptoms 3 or more days each week**
  - **Need for 3 or more doses of rescue inhaler (e.g. salbutamol/Ventolin) each week**
  - **Missed work or school because of asthma within the last 3 months**
  - **Unable to exercise normally or keep up with peers because of symptoms**
- **Anaphylaxis**: history of a severe reaction to tiny amounts of the food, especially in baked goods
- **Adherence**: Unable to commit to daily doses of ladder foods from the step you are working on

If any of these apply to your child, a conversation with your allergist about risks and benefits is recommended BEFORE starting on the ladder.

**Red flag warnings - You should contact your allergist for further direction**

- New diagnosis of asthma or a hospital visit for breathing problems
- If your child has asthma that is not well controlled (see above)
- Reactions worse than mouth itch 3 or more times per week to their ladder dose
- If your child has had anaphylaxis to their dose of ladder food

**What if I can’t get hold of my allergist, and I’m worried about my child continuing with a food ladder?**

**Stop the ladder** if there are red flags described above until you’ve had a chance to meet with your allergist.
